# Recommendations for malaria prevention in moderate to low risk areas: travellers’ choice and risk perception

**DOI:** 10.1186/s12936-015-0654-y

**Published:** 2015-04-01

**Authors:** Rachel Voumard, Delphine Berthod, Clotilde Rambaud-Althaus, Valérie D’Acremont, Blaise Genton

**Affiliations:** Travel Clinic, Department of Ambulatory Care and Community Medicine, University of Lausanne, Rue du Bugnon 44, Lausanne, 1011 Switzerland; Infectious Diseases Service, Department of Medicine, Centre Hospitalier Universitaire Vaudois and University of Lausanne, Lausanne, Switzerland; Swiss Tropical and Public Health Institute and University of Basel, Basel, Switzerland

## Abstract

**Background:**

The considerable malaria decline in several countries challenges the strategy of chemoprophylaxis for travellers visiting moderate- to low-risk areas. An international consensus on the best strategy is lacking. It is essential to include travellers’ opinions in the decision process. The preference of travellers regarding malaria prevention for moderate- to low-risk areas, related to their risk perception, as well as the reasons for their choices were investigated.

**Methods:**

Prior to pre-travel consultation in the Travel Clinic, a self-administered questionnaire was given to travellers visiting moderate- to low-risk malaria areas. Four preventive options were proposed to the traveller, i.e., bite prevention only, chemoprophylaxis, stand-by emergency treatment alone, and stand-by emergency treatment with rapid diagnostic test. The information was accompanied by a risk scale for incidence of malaria, anti-malarial adverse drug reactions and other travel-related risks, inspired by Paling palettes from the Risk Communication Institute.

**Results:**

A total of 391 travellers were included from December 2012 to December 2013. Fifty-nine (15%) opted for chemoprophylaxis, 116 (30%) for stand-by emergency treatment, 112 (29%) for stand-by emergency treatment with rapid diagnostic test, 100 (26%) for bite prevention only, and four (1%) for other choices. Travellers choosing chemoprophylaxis justified their choice for security reasons (42%), better preventive action (29%), higher efficacy (15%) and easiness (15%). The reasons for choosing stand-by treatment or bite prevention only were less medication consumed (29%), less adverse drug reactions (23%) and lower price (9%). Those who chose chemoprophylaxis were more likely to have used it in the past (OR = 3.0 (CI 1.7-5.44)), but were not different in terms of demographic, travel characteristics or risk behaviour.

**Conclusions:**

When travelling to moderate- to low-risk malaria areas, 85% of interviewees chose not to take chemoprophylaxis as malaria prevention, although most guidelines recommend it. They had coherent reasons for their choice. New recommendations should include shared decision-making to take into account travellers’ preferences.

**Electronic supplementary material:**

The online version of this article (doi:10.1186/s12936-015-0654-y) contains supplementary material, which is available to authorized users.

## Background

For non-immune travellers or migrants, the strategy of malaria prevention depends on the risk within the visited area, the season and the duration of stay [1], but also on the policy of the country from where the traveller sought advice before departure [2]. Although all countries agree on prescribing chemoprophylaxis for high-risk endemic regions, recommendations for moderate- to low-risk areas, usually defined as a risk of one infection per 10,000 travellers [3], are variable [4-8]. Switzerland, Austria, Sweden, The Netherlands, and Germany recommend stand-by emergency treatment and bite prevention [9,10] for these regions, whereas the majority of other countries recommend chemoprophylaxis. The Center for Disease Control recently replaced the term ‘stand-by emergency treatment’ with ‘reliable supply regimen’, considering its use in exceptional circumstances only and more as an adjuvant than an alternative to chemoprophylaxis [11].

In these moderate- to low-risk areas, taking chemoprophylaxis exposes travellers to a higher risk of severe adverse drug reactions than actually being affected by malaria [12-14]. The frequency of mild to moderate adverse drug reactions varies from 32-45% [15], underlining the fragile balance between risks and benefits. The rather low adherence of travellers to chemoprophylaxis (30-50%) adds to the controversy about its relevance [16].

Little is known about the preference of travellers in term of malaria prevention. Senn *et al.* highlighted the importance of the price in travellers’ choice of one or other medicine for chemoprophylaxis [17]. However, to the knowledge of the authors, no study has addressed the question of what would be the travellers’ choice in terms of malaria prevention measures for moderate- to low-risk areas, should alternatives be proposed to them. In line with the current momentum of shared decision-making [18], the present study aims at better understanding travellers’ aspirations. The primary objective was to evaluate the personal preference of travellers visiting moderate- to low-risk malaria areas, depending on their perception of risk. The secondary objective was to investigate the reasons for their choice, and correlate it to their sociodemographic profile and risk behaviours.

## Methods

The Travel Clinic in Lausanne is part of the Department of Ambulatory Care and Community Medicine linked to the University Hospital. Health professionals (physicians and nurses) perform around 10,000 pre-travel consultations per year. All travellers attending the Travel Clinic for a pre-travel consultation were screened for eligibility by a research student. Included subjects were adults without pre-travel consultation in the previous year who were planning to visit moderate- to low-risk malaria areas, as defined by the World Health Organization (WHO) [8] with adaptation by the Swiss Federal Office of Public Health (FOPH) [19].

Prior to the pre-travel consultation, four study documents were given to each traveller: a informed consent form, a questionnaire, a table describing the four different malaria prevention methods (see Additional file 1), and a travel-related malaria risk scale. The questionnaire included questions about the sociodemographic profile and the travel pattern. Ten sociodemographic variables were collected: gender, age, country of origin, occupation (categorized according to the Occupational Classification from the International Labour Organization (CITP-08)), co-morbidities, usual treatments (including contraceptive pill), risk behaviours (alcohol consumption according to the National Institute on Alcohol Abuse and Alcoholism, active tobacco use and active or past drug consumption, including cannabis), dependent child or person at home, anti-malarial medication kept at home or used before (as prophylaxis or treatment). Also, seven variables concerning the type of travel were collected: destination (subcontinent according to UN classification [20]), duration of stay, reason for travel (tourism, visiting friends and relatives, humanitarian, business, expatriate), travelling companions (couple, alone, group organized by a travel agency, group outside a travel agency, family), rural/urban areas (rural for < two weeks, rural for ≥ two weeks, only urban, urban and rural, don’t know), and delay to access medical care (<24 hr, ≥24 hr, don’t know).

**Figure 1:**
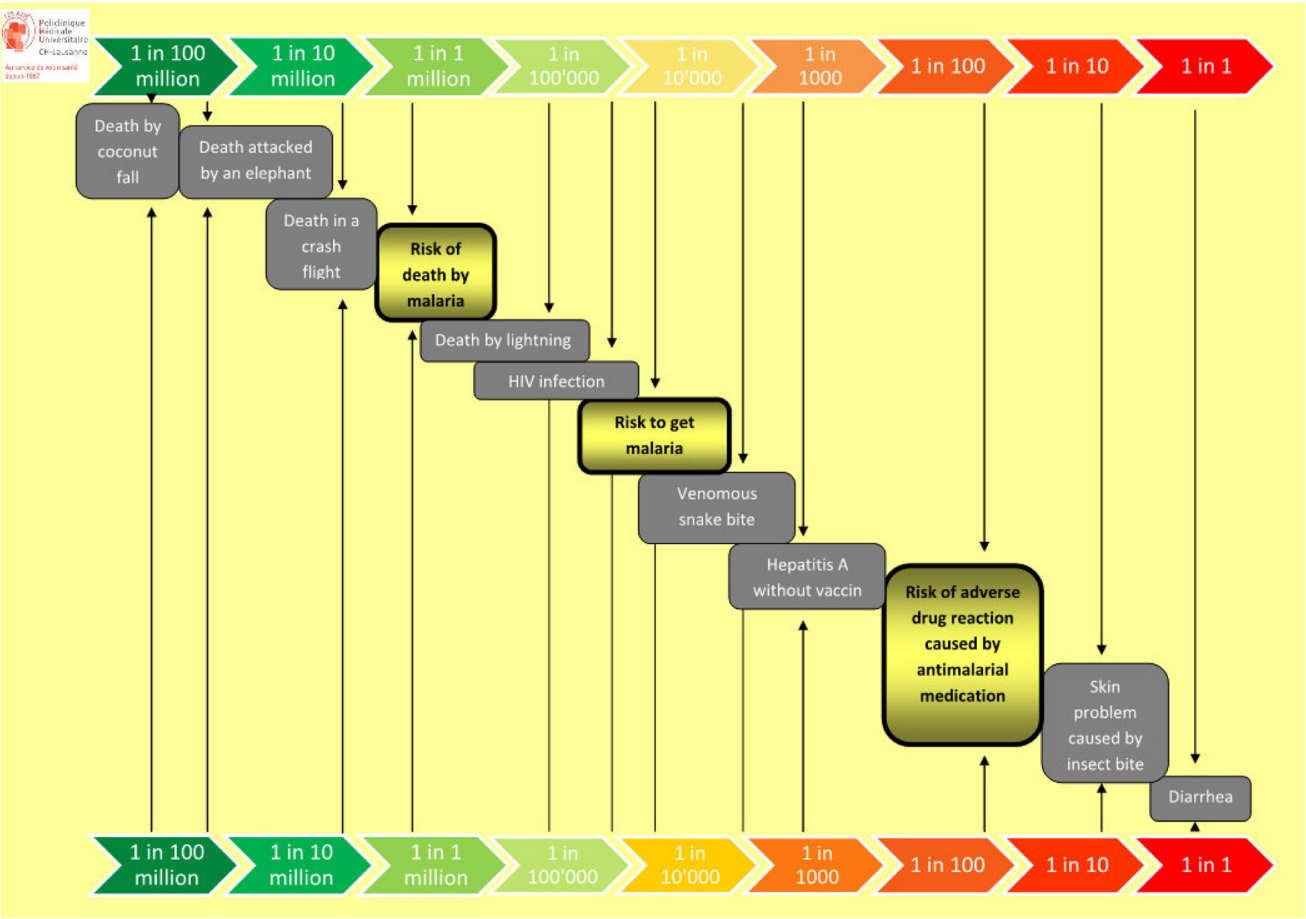
**Travel-risk related scale, derived from the Paling Palette, Risk Communication Institute.** [The 1:10 000 risk of malaria illustrated in the figure describes the risk of malaria in moderate- to low-risk areas (WHO Malaria report 2011)].

The traveller had to choose a malaria prevention method and explain the reasons for the choice, on the basis of a table describing four different options: 1) bite prevention only (to wear long clothes, regular application of mosquito repellent, mosquito net, to limit exposition during high risk period); 2) chemoprophylaxis; 3) stand-by emergency treatment; and, 4) stand-by emergency treatment with rapid diagnostic test. This table highlighted the main advantages and disadvantages of each preventive strategy, and indicated its cost for a two-week period (see Additional file 1: Table A). In addition, the subject received a figure illustrating the risk of malaria and the risk of anti-malarial adverse drug reactions compared to other travel-related risks (Figure 1). The figure was derived from the Paling Palette from the Risk Communication Institute [21]. The Paling Palettes are designed to help healthcare improve the risk communication to patients. This visual support is a decision aid that compares absolute risk instead of relative one [22,23]. A Swiss randomized study showed a higher level of risk perception with Paling Palettes than with others formats [24], yet with differences according to the education level [25]. The traveller was then invited to again choose a malaria preventive measure, should it be given free of charge and give the reasons for choice. Five questions related to malaria (namely incidence, population at risk, treatment, high-risk areas, and clinical manifestations) were also included to assess the traveller’s prior knowledge about the disease.

Following data collection, all travellers received the usual pre-travel advice by a physician or nurse according to the standard procedures used at the Travel Clinic, based on the Swiss FOPH guidelines.

For the qualitative data analysis, the reasons for each choice were classified into 12 categories: travel risk evaluation, security, efficacy, reactivity, easiness, price, medication consumed, adverse drug reactions, medical access, preventive action, diagnostic validation, peer advice/previous experience, and others. All analyses were descriptive. The investigation assessment of risk factors was performed through bivariate analyses to calculate odds ratio and confidence intervals. All data were collected in Microsoft Excel and analysed using Epi Info (CDC version 7.0). The protocol was approved by the ethical review board of the University of Lausanne (No 441/12).

## Results

**Figure 2:**
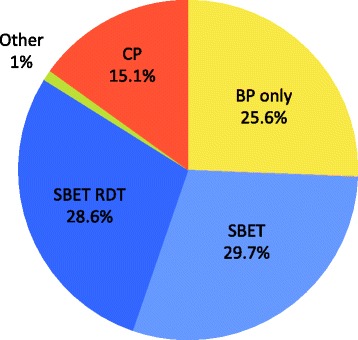
**Travellers choices regarding preventive strategies against malaria (n=391).** CP= Chemoprophylaxis, BP only= Bite prevention only, SBET= Stand-by emergency treatment, SBET RDT= Stand-by emergency treatment with rapid diagnostic tests, Other= CP+SBET+/- RDT.

**Figure 3:**
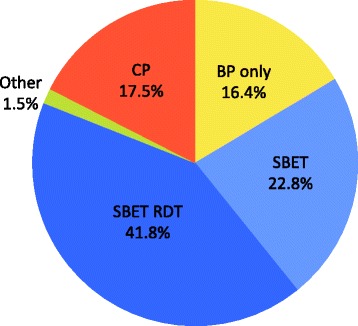
**Travellers choices regarding preventive strategies against malaria if prevention is free (n=391).** CP= Chemoprophylaxis, BP only= Bite prevention only, SBET= Stand-by emergency treatment, SBET RDT= Stand-by emergency treatment with rapid diagnostic tests, Other= CP+SBET+/- RDT

A total of 428 questionnaires were collected between December 2012 and December 2013, and 391 (91%) have been included. Thirty-seven (9%) travellers were then excluded for the following reasons: destination to malaria high or non-endemic areas (13); pre-travel consultation in the previous six months (15); informed consent not signed or dated (6); and, age under 18 years (3). The sociodemographic data and the travel profile are summarized in Tables 1 and 2. The 391 included travellers had a median age of 36 years; 65% (255/391) visited the Asian continent; 63% (247/391) for a duration between two and three weeks. The choice by travellers of malaria prevention when taking into account the price is shown in Figure 2: 15% (59/391) chose chemoprophylaxis (CP), 30% (116/391) stand-by emergency treatment (SBET), 29% (112/391) stand-by emergency treatment with rapid diagnostic test (SBET RDT), 26% (100/391) bite prevention only (BP), 1% (4/391) CP + SBET or CP + SBET RDT. When asked which method he/she would choose if it was free of charge (Figure 3), 76% (295/390) of travellers kept to their initial choice; 17% (68/391) chose CP (+2% when compared to first choice); 23% (89/391) SBET (-7%); 42% (163/391) SBET RDT (+13%); 16% (64/391) BP (-9%); and, 2% (6/391) CP + SBET RDT (+1%) (one did not choose). Among the 100 travellers who had chosen BP when taking into account the price, 34% (34/100) changed to SBET or SBET RDT and 9% (9/100) changed to CP when free of charge.

**Table 1:** **Socio-demographic characteristics of the 391 travellers included**

**Socio-demographic characteristics**	**Frequency %**
Gender female	54% (212)
**Age category (years)**	
18-25	25%(98)
26-30	26% (101)
31-40	22% (86)
41-50	10% (41)
>50	17% (65)
**Country of origin**	
Switzerland (CH)	67% (263)
Out of CH, endemic for malaria	4% (17)
Out of CH, non endemic for malaria	22% (85)
Unknown	7% (26)
**Occupation**	
Managers and intellectual formation	28% (107)
Intermediate training	11% (44)
Administrative or technical training	28% (111)
Farmers, workers, artisans	4% (17)
Students	17% (67)
Jobless, pensioners or unknown	12% (45)
Co-morbidities	13% (52)
Usual treatment (including pill)	24% (95)
**Risk behaviours (OH, tobacco, drugs)**	
Having 1 risk behaviour	26% (101)
Having 2 risks behaviours	12% (47)
Having all 3 risks behaviours	3% (13)
Dependent child or person at home	12% (45)
Anti-malarial medicine kept at home	15% (59)
Anti-malarial medicine used before	
as prophylaxis or treatment	27% (105)

**Table 2:** **Travel characteristics of the 391 travellers included**

**Travel characteristic**	**Frequency %(n)**
**Destination**	45% (177)
South-Eastern Asia	20% (78)
Southern Asia	24% (94)
South America	24% (94)
Carribbean and Central America	4%(15)
Africa (United Republic of Tanzania only)*	5% (20)
Round the world	2% (7)
**Duration of stay**	
1 week	6% (25)
2-3 weeks	63% (247)
4-6 weeks	12% (48)
6-12 weeks	5% (21)
>3 months	13% (50)
**Reason of travel**	
Tourism	83% (324)
Visiting friends and relatives (VFR)	9% (35)
Humanitarian	3% (13)
Business	3% (13)
Expatriate	1% (3)
**Travelling companions**	
Couple	42% (166)
Alone	20% (77)
Group organized by a travel agency	17% (67)
Group outside a travel agency	11% (44)
Family	8% (32)
Unknown	1% (5)
**Rural/urban areas**	
Rural for <2 weeks	29% (114)
Rural for ≥ 2 weeks	18% (72)
Only urban	14% (53)
Urban and rural	8% (30)
Don’t know	31% (121)
**Delay to access medical care**	
<24h	21% (84)
≥24h	18% (71)
Don’t know	58% (228)
Unknown	2% (8)
**Delay to access medical care**	
0-1	25% (96)
2-3	51% (200)
4-5	24% (24)

*All these travellers were visiting Zanzibar with a stop-over in Dar es Salaam. Zanzibar is known to have moderate to low endemicity, and is, therefore, the only area in sub-Saharan Africa where stand-by treatment rather than prophylaxis is recommended according to the Swiss Federal Office of Public Health recommendations.

**Figure 4:**
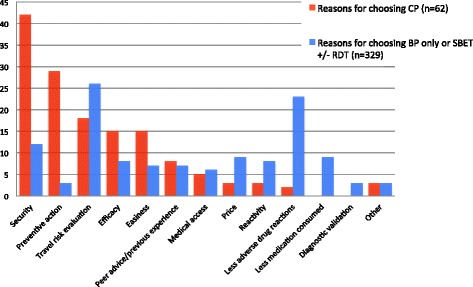
**Travellers reasons for choosing chemoprophylaxis or another preventive strategy (n=391).** CP= Chemoprophylaxis, No CP= No Chemoprophylaxis, BP only= Bite prevention only, SBET +/- RDT= Stand-by emergency treatment +/- rapid diagnostic test.

Figure 4 describes the reasons given by travellers for choosing CP and for choosing another preventive strategy (namely SBET, SBET RDT and BP). Those choosing CP mentioned security (42%), preventive action (29%) and both efficacy (15%) and easiness (15%) as reasons, while those who did not choose CP mentioned less medication consumed (29%), less adverse drug reactions (23%) and price (9%). Only the previous use of anti-malarial was significantly associated with CP (*versus* another choice) (OR 3.0 (95% CI 1.7-5.4). For these travellers who already used anti-malarial medicine (as prophylaxis or treatment), 28% (29/105) chose CP (+18% when compared with naive travellers), 32% SBET (+3%), 23% (24/105) SBET RDT (-8%), 16% (17/105) EP (-14%), and 1% (1/105) no prevention. In term of socio-demographic characteristics, those who used anti-malarial medicine in the past were older (median age 42 vs 34 years old) and had a better knowledge of the disease (41% vs 18% of appropriate responses). For travellers having a dependent person at home and those travelling to Tanzania, the OR for CP was borderline: 1.8 (CI 0.8-3.8) and 2.4 (CI 0.9-6.4), respectively. No other sociodemographic or travel characteristic was associated with CP.

## Discussion

When provided with an illustrative figure that compared the risk of malaria, the risk of adverse drug reactions, and other travel-related risks, the travellers chose stand-by emergency treatment with or without rapid diagnostic test in first place (58%) followed by bite prevention only (26%), while only 15% chose chemoprophylaxis. If the preventive measures were free of charge, 76% of the travellers would have chosen the same method. No recommendation on malaria prevention currently includes travellers’ preferences. Considering the growing trend towards shared decision-making in healthcare, the high rate of travellers opting for not taking chemoprophylaxis calls for an adaptation of the guidelines to integrate travellers’ preferences.

The choice made by the traveller was coherent with the reason he/she gave to support his/her preferred option. The 15% of travellers who chose CP mentioned a ‘higher level of security’ and the ‘preventive action’ as main reasons, whereas the 85% of travellers who chose SBET +/- RDT or BP mentioned ‘fewer medication intake’ and ‘fewer adverse drug reactions’. Although not formally studied using a control group, the use of a risk scale inspired by Paling Palette probably helped the traveller to understand and perceive better his/her own risk. These findings indicate that the traveller is able to assess risk according to his/her perception and to weigh his/her own priorities. This is the essence of the shared decision-making process between client and health professional. One of the main challenges of shared decision-making is to create tools to « diagnose preference » [26]. As the author says, this approach insures that patients or travellers get « the care they need and no less, the care they want and no more ». In this relational process, healthcare recognizes the patient’s own expertise, design by experience of illness, social circumstances, attitude to risk, values and preferences [27]. Some travellers prefer to take a high but not severe risk of anti-malarial adverse event, some others prefer a low but severe risk of having a malaria. The healthcare professional requires advanced communication skills to present the risks and the different options, and decision aid can be a tool [28]. In the present study, the travellers having previous experience of anti-malarial use in the past were feeling more comfortable with the medication risk than with the malaria risk as they were more likely to choose CP as prevention compared to the naive travellers.

The traveller’s choice should not be the main criterion to decide which recommendation to propose. However, his/her opinion should be integrated into the decision process, as recommended in the WHO Guidelines Review Committee [29]. For malaria prevention, the consumer’s perspective has never been considered, which could explain the rather low adherence to the recommendations, especially to chemoprophylaxis [15]. SBET is one among several options, but certainly not the sole one. Adding rapid diagnostic tests (RDTs) might improve appropriateness of SBET use when a traveller is febrile. This needs to be investigated further and a study is ongoing in the Travel Clinic to assess travellers’ behaviour and satisfaction with this new diagnostic tool.

Caution should be applied since the present study has been conducted in a country where SBET is recommended for travellers visiting moderate- to low-risk malaria areas. One can thus consider that the people included in the study were familiar with the Swiss malaria prevention policy and more open to taking risks than other countries such as the USA. Also it could reflect the patient-centered perspective, defined by the Commonwealth Fund’s National Scorecard as ‘care delivered with the patient’s needs and preferences in mind’ [30], and where Switzerland is scored second in world ranking of health care systems by the Commonwealth Fund [31].

## Conclusions

The findings of this study call for more consideration of travellers’ opinions and desires when establishing guidelines. As long as information is provided adequately, the traveller can take more responsibility for his/her own health, according to his/her own risk perceptions and beliefs. The latter should not be the only criterion, but one component of an integrated decision process. Such an approach may both increase adherence to malaria preventive measures and raise the credibility of travel medicine advisors across countries.

## Additional file

Additional file 1:
**The main advantages and disadvantages of four preventive strategies.**
