# Supplementary material for: Recommendations for malaria prevention in moderate to low risk areas: travellers’ choice and risk perception
Source: Malar J. 2015 Apr 1;14:139. doi: 10.1186/s12936-015-0654-y (PMC4396190; doi:10.1186/s12936-015-0654-y)
Supplement: Additional file 1: — The main advantages and disadvantages of four preventive strategies. [file 12936_2015_654_MOESM1_ESM.docx]

Additional file 1:

**The main advantages and disadvantages of four preventive strategies**
